# Long non-coding RNA panel as a molecular biomarker in glioma

**DOI:** 10.1186/s43046-021-00090-4

**Published:** 2021-10-25

**Authors:** Abdol Ali Ebrahimi, Hasan Ashoori, Farnaz Vahidian, Iman Samiei Mosleh, Shaghayegh Kamian

**Affiliations:** 1 School of Medicine, Shahid Beheshti University of Medical Sciences, Tehran, Iran; 2 Baqiyatallah University of Medical Sciences, Tehran, Iran; 3 Faculty of Art and Sciences, Yazd University, Yazd, Iran; 4 University of Tehran Institute of Biochemistry and Biophysics, Tehran, Iran

**Keywords:** Brain neoplasm, RNA, Long noncoding, Prognosis, Biomarkers

## Abstract

**Background:**

Glioma is one of the most malignant brain tumors, accounting for about half of the gliomas that occur in central nervous system (CNS), originates from the glial tissue of the brain. The aim of the present study was to determine the expression levels of 5 lncRNAs (MDC1-AS1, HOXA11-AS, MALAT1, CASC2, ADAMTS9-AS2) in patients with high-grade glioma in comparison with low grade glioma.

**Methods:**

This was a retrospective study which determined molecular biomarker on pathologic glioma samples. We examined 100 patients’ pathologic block which consisted of 50 pathology samples of high-grade glioma (case group) and control group consisted of 50 pathology samples of low-grade glioma. This research was performed using real time polymerase chain reaction (PCR) technique.

**Results:**

The results showed that the expression of ADAMTS9-AS2 and HOXA11-AS genes significantly increased with increasing tumor grade. Also the expression of CASC2 gene significantly decreased with increasing tumor grade.

**Conclusions:**

It was concluded that ADAMTS9-AS2 and HOXA11-AS and CASC2 are promising lncRNA markers in prognosis of glioma.

## Background

Brain tumors are common causes of death among all malignancies and the second most common brain disorders after cerebral stroke [1]. Primary cerebral tumors rise from the brain and can infiltrate throughout the tissue; nevertheless, they are not always cancerous or malignant [2]. Malignant brain tumors may either originate in the brain or spread from other parts of the body to the brain, which is a potentially fatal condition. The rate of tumor growth and spread, as well as the presence of specific cells within the tumor are the criteria used to ascertain disease severity or tumor grade [3].

Glioma comprises 30% of primary brain tumors, and according to the WHO classification, they can be categorized into four grades (i.e., I to IV) based on histopathologic features [4]. Among these four grades, glioblastoma (GBM) has the poorest prognosis and the lowest survival rate, Despite available treatments including surgery, radiotherapy, and chemotherapy; therapeutic and clinical outcomes are still unsatisfactory [5, 6].

One of the factors that can help to increase the effectiveness of treatment is to better understanding of the molecular pathogenesis and genetic basis of glioma. Previous studies have shown that long non-coding RNAs (lncRNA) showed dysregulation and dysfunction in many malignant human tumors such as colorectal, prostate, bladder, liver, and brain [7, 8]. Recent evidence indicates that lncRNAs probably play an important role in the pathogenesis of glioma [9]. For example, these molecules can modulate cellular proliferation and apoptosis, leading to tumorigenesis. Also, studies show that the abnormal expression of lncRNAs leads to poor prognosis, especially in GBMs. Therefore, lncRNAs seem to be applicable as potential diagnostic markers or therapeutic targets [10].

According to the important role of lncRNAs in the pathogenesis of gliomas in regulating cellular proliferation, apoptosis, and tumorigenesis and also clinical outcomes and prognosis in glioma, this study was designed to investigate the expression profile of several lncRNAs.

## Methods

This was a retrospective study that determined molecular biomarkers on pathologic glioma samples. In this study, a hundred astrocytoma pathologic paraffin-embedded tissues were reviewed. These patients had brain mass and had surgery. Fifty of them were low-grade glioma (grades I and II) and 50 were high-grade glioma (grades III and IV). All patients signed an informed consent form before surgery and agreed to their tissue samples being used in the research project. All ethical requirements were observed during sample collection. The samples were obtained by surgeons in the operation room and stored in a biobank after being confirmed by pathological examination. Inclusion criteria were a definitive diagnosis of glioma by a neurosurgeon and a radiologist before surgery and confirming pathological features and determining tumor grade by a pathologist. We evaluated the samples of adult patients ranging from 14 to 80 years old. Fibrillary astrocytoma was excluded.

Tumor tissues were transferred into 1.5 mL sterile DNAse/RNAse free microtubes, deparaffinized, and stored. RNA extraction was performed using a RNA extraction kit (MN Co, Germany, Cat No: 740304). Selected lncRNAs for the present study were MDC1-AS1, HOXA11-AS, MALAT1, CASC2, and ADAMTS9-AS2. Primer sequences used in this study to amplify target genes by real-time PCR method showed in Table 1. TaqMan probe real-time PCR method used for specific amplification of lncRNAs and expression levels of these genes were compared using U6 as reference gene.

**Table 1:** Primers used in this study to amplify target genes

Name of lncRNA	Primer sequence (3′-5′)
**MDC1-AS1**	F: -TCCCAGATGTGCCAAAGTCAG R: -AGCAACCCCAGTTGTCATTC
**HOXA11-AS**	F: TGCCAAGTTGTACTTACTACGTC R: GTTGGAGGAGTAGGAGTATGTA
**MALAT1**	F: ATGCGAGTTGTTCTCCGTCT R: TATCTGCGGTTTCCTCAAGC
**CASC2**	F: TACAGGACAGTCAGTGGTGGTA R: ACATCTAGCTTAGGAATGTGGC
**ADAMTS9-AS2**	F: CAGAAGGGGCTTGGTTGG R: TCGTGTTCCTACCCTATTTTGA

To analyze the qRT-PCR result data, the expression of each gene were normalized with as endogenous housekeeping gene, U6, and regular ΔΔct method was used to calculate gene expression fold change. After normalization of the data with log2 the regular unpaired sample T test was used to compare expression of each candidate gene in test group (glioma) and control (normal) group.

## Results

### Demographic features of the studied population

Demographic information of assessed patients is mentioned in Table 2. The mean age of patients was 43.70 ± 16.416 years.

**Table 2:** Demographic and clinical information of studied patients

Variables		Mean ±SD (range)	Percentage
Age (years)		43.70 ± 16.416 (40–75)	
Grade	I		5.3 %
	II		31.9 %
	III		16.7 %
	IV		46.1 %

### Gene expression findings

#### MDC1-AS1 gene expression

There was no statistically significant difference in MDC1-AS1 gene expression between low- and high-grade tumors (*p* = 0.63). The relative expression of this gene (fold change) was − 0.2 in high-grade (III and IV) respective to low-grade (I and II) tumor tissues.

#### HOXA11-AS gene expression

Our findings showed that the expression of HOXA11-AS gene significantly increased in high-grade tumors (*p* = 0.01). The relative expression of this gene in grade III and IV samples was three folds higher compared to grade I and II tumors.

#### MALAT1 gene expression

There was no association between the expression of MALAT1 gene and tumor grade (*p* = 0.45). The relative expression of this gene was 0.3-fold higher in high-grade (III and IV) compared to low-grade (I and II) samples.

#### CASC2 gene expression

The results showed that the expression of CASC2 gene significantly decreased with increasing tumor grade (*p*=0.01). The relative expression of this gene was 0.5-fold lower in high-grade (III and IV) compared to low-grade (I and II) tumor samples.

#### ADAMTS9-AS2 gene expression

Our results showed that the expression of ADAMTS9-AS2 gene significantly increased with increasing tumor grade. The relative expression of this gene was 4.1-fold higher in grade III and IV compared to grade I and II tumors.

**Fig. 1:**
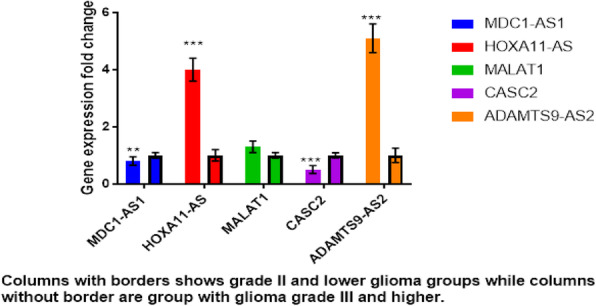
Gene expression fold changes

Changes in the expression of assessed genes in tumor tissues have been shown in Table 3. Expression levels of lncRNAs was compared using Mean of fold change (ratio of grades III and IV/grades I and II). *P* value > 0.05 considered significant (Fig. 1).

**Table 3:** The expression of target genes in low- and high-grade glioma tumor tissues

Name of lncRNA	Expression change	Mean of fold change (ratio of grades III and IV/grades I and II)	*P* value
MDC1-AS1	Downregulated	0.2	0.63
HOXA11-AS	Overexpressed	3	< 0.01
MALAT1	Overexpressed	0.3	0.45
CASC2	Downregulated	0.5	< 0.001
ADAMTS9-AS2	Overexpressed	4.1	< 0.001

### Literature review and lncRNA prioritization

Glioma related articles were found in pubmed database by searching “Glioma and lncRNA” phrase. After reviewing all related articles, six are lncRNAs including, MALAT1, CASC2, MAMDC2-AS1, ADAMTS9-AS2, and HOXA11.

### Microarray data analysis and co-expression network design

The data (GSE104267) were retrieved from NCBI-GEO database and comprised of three normal brain tissues and six glioma tumor tissues. After data cleaning and preparation, all the data were normalized using normalize-quantile method. Then the differentially expressed genes between tumor and normal tissues were found using limma package in R. By the application of psych package in R the possible correlation between each pair of differentially expressed genes and the six candidated lncRNAs according to the literature were calculated. The network of correlated genes and the degree and betweenness values of each node were obtained using R, igraph package. Finally the co-expression network were illustrated using Cytoscape V6 software.

**Fig. 2:**
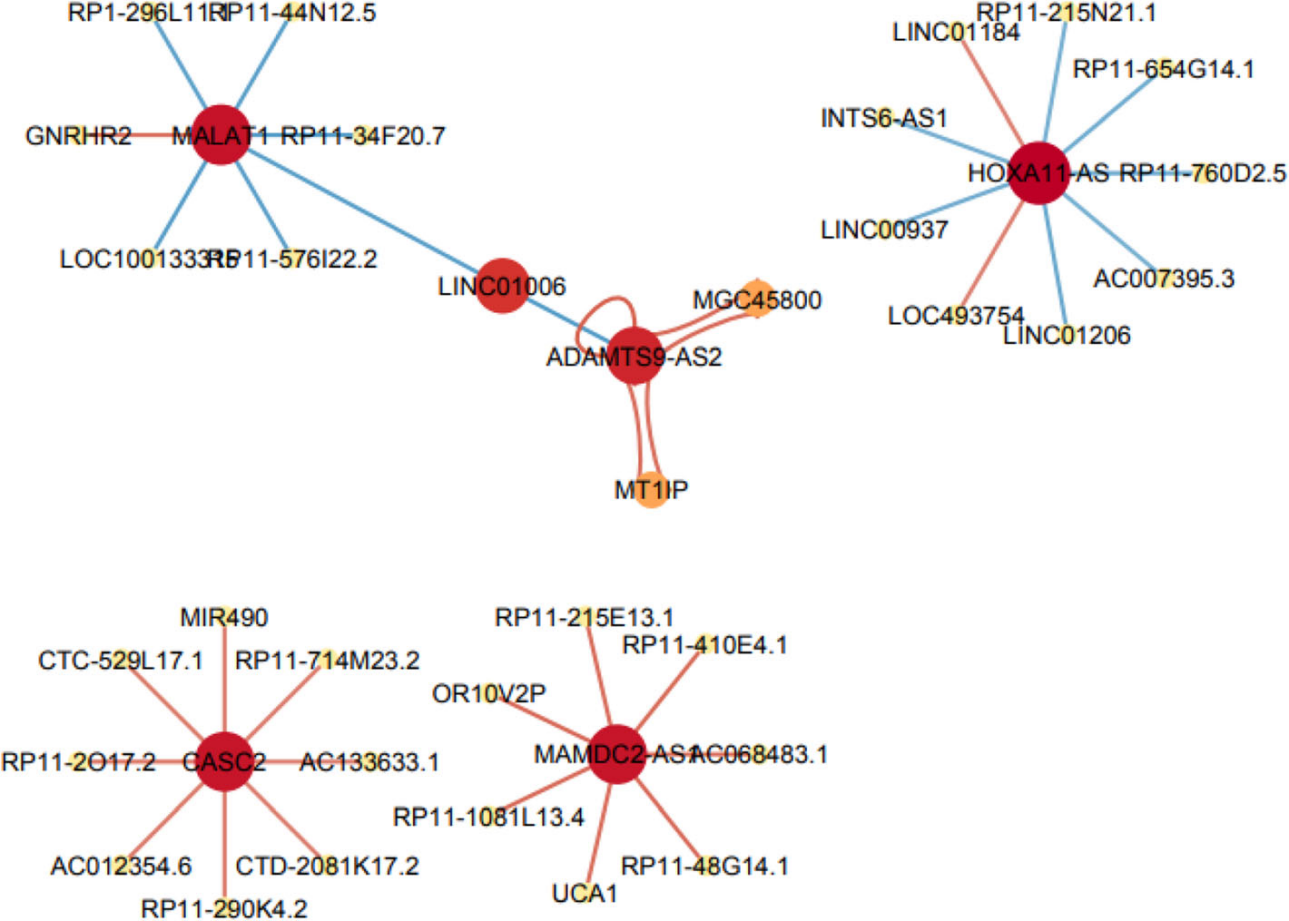
Co-expression network of deferentially expressed and six candidated lncRNAs in glioma tissues in comparison with the normal brain tissues

Figure 2 shows the co-expression network of deferentially expressed and six candidated lncRNAs in glioma tissues in comparison with the normal brain tissues. Each node represent a differentially expressed lncRNA and each edge represent the correlation. Darker nodes with larger shapes shows higher degree and betweenness and darker edge shows higher correlation (Table 4).

**Table 4:** The degree of betweenness centrality and rank-stat values of each candidated lncRNAs in the network

Gene	Degree	Betweenness	Rank_stat
HOXA11-AS	9	36	38.5
MALAT1	7	39	37
CASC2	8	28	36.75
MAMDC2-AS1	8	28	36.75
ADAMTS9-AS2	8	17	35.5

## Discussion

Glioma is the most common type of brain malignancy. The diagnosis of gliomas is generally problematic due to high heterogeneities between various glioma subtypes, as well as within tumors of a unique subtype. Dysregulated expression of lncRNAs can trigger the development and progression of several cancers including gliomas [11]. LncRNAs are able to regulate gene expression through various mechanisms; however, many of these mechanisms have not been fully defined yet. LncRNAs have essential roles in cell fate through regulating gene expression at transcription, post-transcription, and epigenetic levels. Epigenetic changes and mutations can alter the expression of lncRNAs transforming them into cancerous transcripts. Different expression patterns of lncRNAs have been described in healthy and malignant cells, as well as in patients with different clinical features. Regarding this, lncRNAs can be used as specific diagnostic and prognostic markers in gliomas [12].

LncRNAs are a significant component of the genetic determinants of cancer, which, in conjunction with other agents, play a decisive role in controlling the growth, division and differentiation of the cell. Due to their complex 3D structures and ability to bind to DNA, RNA, and proteins, lncRNAs can regulate various cellular processes [13, 14]. These molecules are mainly present in the nucleus and are involved in regulating gene expression, DNA replication, as well as the formation of nuclear structures. Fewer lncRNAs are present in the cytoplasm and participate in the regulation of post-transcription gene expression, protein trafficking, transferring mRNAs between the nucleus and cytoplasm, and the regulation of the activity of proteins [15]. The number of lncRNAs used as biomarkers in diagnosis and prognosis is increasing every year, and some have been approved for clinical use. Some lncRNAs can act as a biologic marker or as a prognostic factor only all lncRNAs play a determining role in diagnosis and prognosis, on cellular processes that create the cancer phenotype, such as proliferation, invasion and survival.

In a study conducted by Yue H et al., LncRNA MDC1-AS was examined as a regulator of cell proliferation in gliomas. The results of that study showed that the expression of this lncRNA in glioblastoma tumor samples has a significant decrease compared to normal tissue [16].

In a systematic review and meta-analysis conducted by Zhou et al. In a total, 40 studies, urothelial carcinoma associated 1 (UCA1 expression was positively associated with tumor size and grade (*P* < 0.001). Also, In the prognostic meta-analysis, high metastasis-associated lung adenocarcinoma transcript 1 (MALAT1) expression could predict poor overall survival (OS) in patients with glioma, with a pooled HR of 2.32 (95% CI: 1.64–3.27, *P* < 0.001) [17].

In another study the expression of AGAP2-AS1, LINC01198, and MIR155HG increased with tumor grade, but TPT-AS1 was a protective lncRNA which had greater expression in the low-grade glioma [18].

Alja Zottel et al. focused on the five most aberrantly expressed lncRNAs in glioblastoma including nuclear MALAT1 (also known as nuclear-enriched abundant transcript 2 (NEAT2)), MEG, HOTAIR, H19, and Colorectal Neoplasia Differentially Expressed (CRNDE). They concluded that for determining new mechanisms of temozolamide (TMZ) resistance by underlying molecular mechanisms of action might be searched in lncRNAs that impact upon increased expression of EMT markers. Careful selection of lncRNAs for evaluation of their roles in vivo is essential to determine the prognosis and response to standard chemotherapy agents [19].

A study conducted by Mahinfar et al. focused on the roles of lncRNAs in the development of multidrug resistance (MDR) against some chemotherapy agents including TMZ in glioblastoma. In this study they evaluated MALAT1, H19, Small nucleolar RNA host gene 12 (SNHG12), ADAM metallopeptidase thromGenes bospondin type 1 motif 9 antisense RNA 2 (ADAMTS9-AS2). They concluded mechanisms of miRNAs in MDR in glioblastoma have been contributed to several major pro-oncogenic signaling pathways [20]. All the studies reviewed here are summarized in Table 5.

**Table 5:** Summary of studies about lncRNA

Author (year)	Title	Area of research	Results
Yue H et al. (2016)	MDC1-AS, an antisense long noncoding RNA, regulates cell proliferation of glioma	LncRNA MDC1-AS	Expression of lncRNA in glioblastoma tumor samples has a significant decrease compared to normal tissue.
Zhou Q. et al. (2018)	lncRNAs as potential molecular biomarkers for the clinicopathology and prognosis of glioma: a systematic review and meta-analysis	1-UCA1 2-MALAT1	1- Expression was associated with tumor size and tumor grade. 2- Expression could predict poor overall survival (OS) in patients with glioma.
Wang W. et al (2016)	LncRNA profile study reveals four-lncRNA signature associated with the prognosis of patients with anaplastic gliomas	1- AGAP2-AS1 2- LINC01198 3- MIR155HG 4- TPT1-AS1	-The expression of 1, 2, and 3 was increased in high-grade tumors. -4 was decreased in high-grade tumors.
Zottel A. et al (2020)	Coding of Glioblastoma Progression and Therapy Resistance through Long Noncoding RNAs	1- MALAT1 2- MEG 3- HOTAIR 4- H19 5- CRNDE	1- Downregulated in glioma 2- Downregulation in human glioma cell lines compared with normal astrocytes 3- Overexpressed in GBM and higher level in serum of patients with glioma specially glioblastoma 4- Expression is significantly increased in glioblastoma tissue, attributed to high tumor grade, and is predictor of poor prognosis. 5- Significant independent prognostic factor for OS and expression correlates with larger tumor size and more recurrence
Mahinfar P. et al. (2021)	Long Non-Coding RNAs in Multidrug Resistance of Glioblastoma	lncRNA’s contribution to multidrug resistance (MDR)	Mechanisms related to MDR: - Targeting MDR transporters - Modulating apoptosis - Targeting DNA repair machinery - Controlling cancer stem cells - Regulating epithelial mesenchymal transition

Our results showed that the expression of ADAMTS9-AS2 and HOXA11-AS genes significantly increased with increasing tumor grade. Also the expression of CASC2 gene significantly decreased with increasing tumor grade. We concluded that ADAMTS9-AS2 and HOXA11-AS and CASC2 are promising lncRNA markers in prognosis of glioma.

## Conclusions

It was concluded that ADAMTS9-AS2 and HOXA11-AS and CASC2 are promising lncRNA markers in prognosis of glioma. The problem with this study were the expenses and gathering the appropriate samples. It seems that if another study could be planned with more samples, the results could be confirmed more precisely.

## Data Availability

The datasets generated and/or analyzed during the current study are not publicly available, but are available from the corresponding author on reasonable request.

## Abbreviations

CNS: Central nervous system
lncRNAs: Long non-coding RNA
GBM: Glioblastoma
DNA: Deoxyribonucleic acid
PCR: Polymerase chain reaction
TMZ: Temozolamide
